# Salivary Biomarkers in Crohn’s Disease and Ulcerative Colitis: A Scoping Review and Evidence Map

**DOI:** 10.3390/ijms262211195

**Published:** 2025-11-19

**Authors:** Karina Oliveira Santos, Ligia Yukie Sassaki, Maiara Brusco De Freitas, Julio Pinheiro Baima, Murilo Henrique Faria, Anna Luisa Bizotto, Júlia Pardini Benício, Ana Carolina Magalhães

**Affiliations:** 1 Department of Biological Sciences, Bauru School of Dentistry (FOB), São Paulo University (USP), Bauru 17047-002, SP, Brazil; 2 Department of Internal Medicine, Medical School, São Paulo State University (UNESP), Campus Botucatu, Botucatu 18618-970, SP, Brazil; 3 Center for Molecular Prediction of Inflammatory Bowel Disease (PREDICT), Department of Clinical Medicine, Aalborg University (AAU), 2450 Copenhagen, Denmark; 4 Human Movement Research Laboratory (MOVI-LAB), Department of Physical Education, Faculty of Sciences, São Paulo State University (UNESP), Bauru 17033-360, SP, Brazil

**Keywords:** biomarkers, saliva, Crohn’s disease, ulcerative colitis, oral health

## Abstract

Salivary biomarkers have been explored as potential non-invasive tools for the diagnosis and monitoring of inflammatory bowel diseases (IBD), including Crohn’s Disease (CD) and Ulcerative Colitis (UC). This study presents a scoping review and evidence mapping on the use of saliva as a matrix in which biomarkers can be identified for these conditions. A systematic search of multiple databases and studies was conducted until 28 January 2025, resulting in the selection of 12 relevant articles. The quantified evidence synthesis identified eight molecular and microbial categories. Of these, four classes, including cytokines, microRNAs, calprotectin, and the microbiome, have demonstrated the most consistent potential. Alterations in these biomarkers, such as an increase in the *Prevotella* genus within the microbiome, and elevated PSMA7 levels, may reflect disruptions in intestinal barrier integrity and immune response. However, factors such as oral health status, hygiene habits, and medication must be carefully considered. Therefore, further clinical research is essential to validate specific biomarkers.

## 1. Introduction

The use of saliva molecular identification has grown in recent years, notably when it comes to the diagnosis and monitoring of diseases [1,2]. As a biological fluid, saliva consists of water, electrolytes, proteins, and other biomolecules [1,3], and is predominantly secreted by the major salivary glands and numerous minor glands [1,3]. Saliva secretion is mainly controlled by the autonomic nervous system, and modulated by mechanical, gustatory, olfactory, and emotional stimuli [3]. The choice of collecting stimulated or unstimulated saliva is critical for biomarker analysis, because the type of stimulus can significantly influence the concentrations of specific components (such as proteins and water content) and the contribution of each salivary gland to the final sample [3].

New technologies known as omics, such as genomics, transcriptomics, proteomics, and metabolomics [1], have enabled the use of saliva to replace or complement more invasive traditional tests, such as those involving blood, urine, and cerebrospinal fluid [2,4]. The term salivaomics can be associated with five potential diagnostic molecules: proteins, mRNA, microRNA, metabolic compounds, and microbes [1,5]. Notably, the microorganisms found in saliva could be linked to the gut [6,7]. The gut and oral microbiomes share species, and changes in the oral community can contribute to intestinal inflammation and systemic disease [7,8]. Therefore, analyzing the salivary microbiome is a crucial approach for understanding the pathogenesis, and identifying non-invasive biomarkers for these conditions.

A previous review about oral condition demonstrates that several biomarkers have already been found in saliva [9], including some oxidative stress and lipid peroxidation markers such as myeloperoxidase (MPO) and nitric oxide (NO), as well as pro-inflammatory cytokines, such as IL-6 and TNF-α [10,11,12,13]. These same cytokines may also be associated with systemic conditions such as diabetes, inflammatory bowel disease (IBD), cardiovascular disease, or cancer [9]. Although saliva appears promising, further studies in this area are needed.

IBD, a chronic multifactorial condition of the gut characterized by immune-mediated inflammation, has shown potential links to salivary biomarkers [6]. Examples include Crohn’s disease (CD) and ulcerative colitis (UC) [13]. Szczeklik and colleagues [11] reported higher salivary levels of IL-1β, IL-6, and TNF-α in patients with active CD than in patients with inactive disease and controls. Furthermore, Rezaie and colleagues [10] revealed an increase in TGF-β1 and NO in patients with UC. Both studies used enzyme-linked immunosorbent assay (ELISA) tests. Future studies should consider combining novel omics techniques with already well-established techniques, such as ELISA tests.

Despite the growing number of studies on salivary biomarkers in IBD, such as CD and UC [6,10,11], the effectiveness and applicability of these biomarkers for the diagnosis and monitoring of these conditions remain uncertain [6].

**Figure 1:**
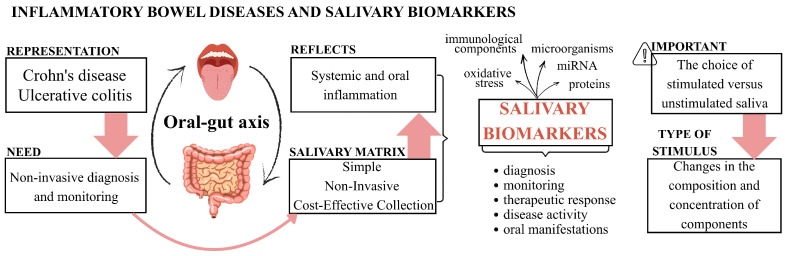
A graphical description of salivary biomarkers in inflammatory bowel diseases. The diagram illustrates the key concepts of the introduction. It begins with the clinical challenge and the need for non-invasive diagnosis and monitoring of Crohn’s Disease (CD) and Ulcerative Colitis (UC). The central concept focuses on the oral-gut axis, which justifies the use of salivary matrix as a simple, non-invasive, and cost-effective collection alternative. The evidence mapping focuses on the classes of salivary biomarkers studied (such as, microorganisms microRNAs, proteins) and the type of biomarker application (such as, diagnosis, monitoring) and highlights the importance of methodological considerations regarding the choice between stimulated versus unstimulated saliva, which affects the composition and concentration of components.

For salivary biomarkers to be incorporated into clinical practice, it is crucial to understand how the studies were conducted, identify gaps in the literature, and assess the clinical response of patients. The unequal expression of miRNAs between CD and UC [14], as well as markers such as TGF-β1 and NO [10], can help distinguish between the two diseases and predict their severity. However, current knowledge remains fragmented, with no consensus on which biomarkers should be used. This review aimed to synthesize the available evidence on potential biomarkers for the identification and monitoring of IBD, as well as for the differentiation between UC and CD, in addition to highlighting existing methodological gaps and Figure 1 briefly illustrates the introduction and aim of this review.

## 2. Materials and Methods

### 2.1. Study Design

This scoping review was conducted in accordance with the Preferred Reporting Items for Systematic reviews and Meta-Analyses extension for Scoping Reviews (PRISMA-ScR) guidelines “https://www.acpjournals.org/doi/10.7326/M18-0850 (accessed on 4 February 2025)”. The review protocol was registered on the Open Science Framework (OSF) “https://doi.org/10.17605/OSF.IO/75MKZ (accessed on 16 January 2025)”, where it is available in full.

### 2.2. Literature Search

The research question was developed using the PCC mnemonic (Population: CD or UC patients; Concept: biomarkers; Context: molecular and microbial analysis using saliva). The search strategy was performed in MEDLINE through PubMed/NCBI (National Center for Biotechnology Information, US National Library of Medicine), Web of Science (Clarivate™), and SCOPUS (Elsevier).

The database search strategy included MeSH search descriptors; biomarkers, saliva, Crohn’s disease and Ulcerative Colitis or alternative terms mixed with Boolean operators OR, AND: “saliva” OR “salivary proteins and peptides” OR “saliva peptide” OR “salivary peptide” OR “saliva peptides” OR “salivary gland protein” OR “saliva protein” OR “salivary protein” OR “saliva proteins” OR “salivary gland proteins” OR “salivary proteins” AND “biomarkers” OR “biomarker” OR “surrogate endpoint” OR “surrogate endpoints” OR “surrogate endpoints” OR “biological markers” OR “biochemical markers” OR “clinical markers” OR “immunological markers” OR “serological markers” OR “surrogate markers” OR “serum markers” OR “viral markers” OR “laboratory markers” OR “serum markers” OR “surrogate result” AND “Crohn’s disease” OR “granulomatous colitis” OR “granulomatous enteritis”, OR “regional enteritis” OR “ileocolitis” OR “regional ileitis” OR “terminal ileitis” OR “ulcerative colitis”.

### 2.3. Eligibility Criteria

The inclusion criteria were as follows: (1) articles published in the English language; (2) abstract available for screening; (3) individuals diagnosed with CD or UC (male or female); (4) involving adult population; (5) published in a peer-reviewed scientific journal until 28 January 2025; and (6) biomarkers present in human saliva. The exclusion criteria comprised (1) studies using pre-clinical models (e.g., animals or cell culture); (2) studies that used other types of samples other than saliva (studies that used saliva and other samples were included); (3) studies that included individuals with other diseases different from CD or UC; (4) abstracts published in conferences; and (5) review articles or book chapters. Moreover, articles without the descriptors cited above in the title, abstract, and/or keywords were excluded.

### 2.4. Study Selection, Data Extraction, and Interpretation

The initial step, data identification and selection, was conducted by K.O.S, after which the references were managed using Mendeley software (Mendeley Reference Manager v2.129.0, Elsevier, London, UK). The data were extracted from the databases in .txt files containing the references of all selected articles and were exported to the Rayyan reference manager “https://www.rayyan.ai (accessed on 28 January 2025)”, where duplicate studies were removed. Data from the included studies were recorded in a standardized Rayyan template. The template was tested by two reviewers (M.H.F. and K.O.S.) to ensure consistency and refine data categories before final extraction. The reviewers (M.H.F. and K.O.S.) examined the titles, abstracts, and keywords. For each included study, the following data were extracted: author, year, country, study design, sample characteristics, biomarkers assessed, and main findings. The charting results were then summarized and/or presented, as they relate to the review questions and objectives. In case of disagreements, a third reviewer (A.L.B.) was consulted. Studies that met the inclusion criteria were subjected to validation and data extraction. During the second and third stages, the evaluators screened the publications using the PCC mnemonic to enhance inter-rater reliability.

Following the data extraction, the collected information (as presented in Table 1) underwent narrative and thematic synthesis. This step involved qualitative grouping of the main findings based on the molecules/microorganisms studied (such as, cytokines, oxidative stress markers, microRNAs, and microbiota), and the key research concepts (e.g., oral-gut axis, oral health status, and analytical methodologies). This thematic approach allowed for a comprehensive discussion of the evidence and the identification of research gaps, ensuring that the results directly addressed the scoping review objective.

### 2.5. Quantified Synthesis and Categorization of Biomarkers

A quantified evidence synthesis matrix was used to synthesize the potential of salivary biomarkers for the diagnosis, monitoring, or therapeutic selection of Crohn’s Disease (CD) and Ulcerative Colitis (UC) (Figure 6).

This matrix classified the findings of each study into predefined molecular groups, based on the following classification criteria and a scoring system based on the total number of studies included (N = 12): The “✓” symbol indicates that the analysis of the respective molecule(s) has been performed and deemed plausible as a biomarker for these conditions. The “X” symbol corresponds to studies that analyzed the molecule but did not identify it as a viable biomarker. The “?” symbol represents situations where the study only mentioned the molecule without methodological clarity, used it as an exclusion criterion, or raised doubts about whether inflammation or oral lesions are a cause or consequence of the disease. The “—” symbol indicates that the molecule was not analyzed in the respective study. Finally, the “*” indicates that, although the study suggests the molecule as a potential biomarker, the interpretation depends on variables such as the disease investigated, inflammatory activity, lesion location and extent, treatment, disease duration and severity, as well as the presence of oral inflammation. Each molecular group was assigned a score ranging from +12 (if all studies indicated the biomarker’s potential) to −12 (if all studies rejected it). Therefore, a higher score suggests a greater potential as a biomarker, while a lower score suggests lower applicability in this context. The score was calculated using the following formula:

$$Score=\;\sum \left(studies\;with\;“✓”\;\right)-\sum \left(studies\;with\;“X”\right)\;$$

## 3. Results

### 3.1. Selection of Sources of Evidence

**Figure 2:**
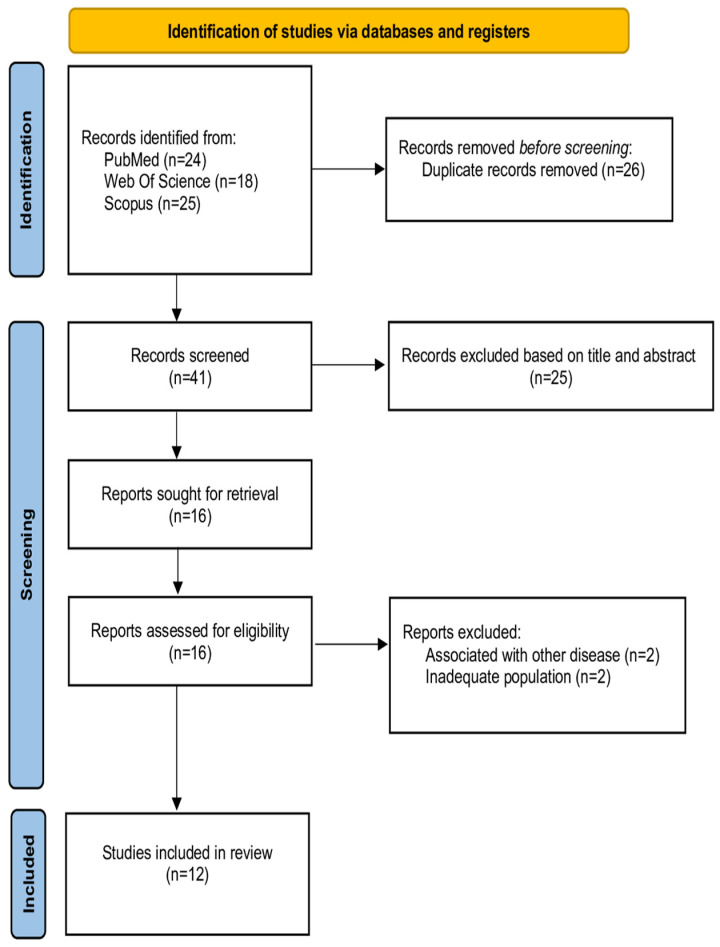
Flowchart of the literature review. This diagram illustrates the flow of information through the phases of the scoping review: Identification, Screening, and Inclusion. It details the number of records identified through database searching and other sources; the removal of duplicates; the initial screening by title and abstract; the full-text assessment for eligibility against the predefined criteria; and the final number of studies included in the review, along with the reasons for exclusion at the full-text stage (e.g., associated with other disease; inadequate population).

Figure 2 demonstrates the flowchart of the literature search.

### 3.2. General and Individual Characteristics of Sources of Evidence

**Figure 3:**
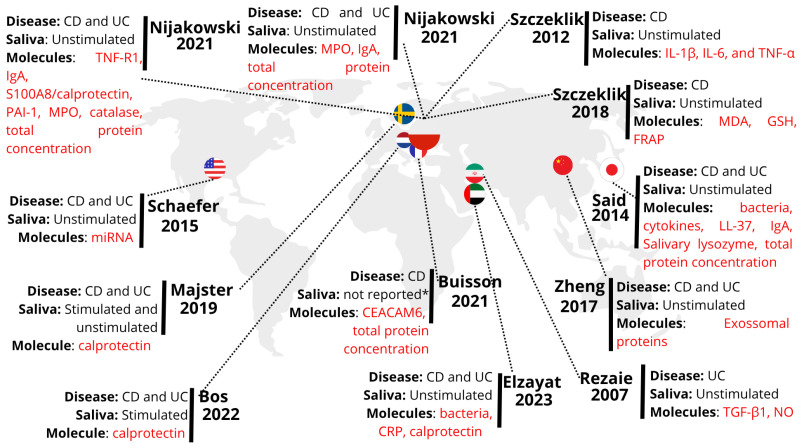
Map showing the 12 included studies, the investigated diseases, type of saliva, and molecules. The circles with flags indicate the country of origin for each study, with circle sizes corresponding to the number of studies conducted by that country. CD: Crohn’s disease. UC: Ulcerative colitis. IgA: Immunoglobulin A. TNF-R1: Tumor Necrosis Factor Receptor Type 1. PAI-1: Plasminogen Activator Inhibitor-1. MPO: myeloperoxidase. miRNA: microRNA. CEACAM6: Carcinoembryonic Antigen Related Cell Adhesion Molecule 6. IL-1β: interleukin IL-1β. IL-6: interleukin 6. TNF-α: anti-tumor necrosis factor α. MDA: malondialdehyde. GSH: glutathione. FRAP: ferric reducing ability of plasma. LL-37: cathelicidin. TGF-β1: Transforming Growth Factor-β1. NO: Nitric Oxide. CRP: C-reactive protein. The symbol “*” in the figure represents the lack of information regarding the explanation of the type of saliva [7,10,11,12,13,14,15,16,17,18,19,20].

The earliest included study was published in 2007 [10]. Figure 3 illustrates the research centers where the included studies were conducted, the investigated diseases, and the type of analysis performed. The research centers were located in nine countries.

### 3.3. Basics Characteristics of Each Study

The individual characteristics regarding the author, objective, groups, sample type, and index used for disease activity classification are represented in Table 1.

**Table 1:** Characteristics of the 12 included studies on salivary biomarkers for Crohn’s Disease (CD) and Ulcerative Colitis (UC). This table summarizes the key features of the sources of evidence, detailing the study author and year, the main objective, the characteristics and number of participants, the diagnostic method used to determine IBD activity, and the type of saliva sample collected for analysis. The following abbreviations and acronyms refer to the diagnostic methods used to assess disease activity: **MTWSI**: modified Truelove-Witts severity index, this is used to categorize the severity of disease into mild, moderate and severe, but yields a numeric score for UC activity between 0 and 21. This index is composite of subjective (number of stools, nocturnal diarrhea, bloody stools, fecal incontinence, abdominal pain, general well-being, and use of antidiarrheal drugs) and objective (abdominal tenderness) questions. **CDAI**: Crohn’s disease activity index, CDAI assess the activity of Crohn’s disease by combining clinical symptoms and laboratory parameters into a quantitative score. The calculation considers factors such as bowel movement frequency, abdominal pain intensity, general well-being, presence of complications, use of antidiarrheal drugs, abdominal mass, hematocrit, and weight variation. The scores classify the disease as remission (<150), mild (150–220), moderate to severe (220–450), and severe (>450). **IOIBD**: International Organization of Inflammatory Bowel Disease index; it is a simplified system for assessing disease activity, considering six clinical parameters: abdominal pain, diarrhea, rectal bleeding, fever, weight loss, and extraintestinal complications, assigning 1 point for each. The score ranges from 0 to 6, classifying the disease as remission/mild (0–1), moderate (2–3), or severe (4–6). **UC-DAI**: Ulcerative Colitis Disease Activity Index; it is an index used to assess the severity of UC, combining clinical and endoscopic parameters. It considers four variables: bowel movement frequency, rectal bleeding, endoscopic appearance of the mucosa, and physician’s global assessment, each scored from 0 to 3, with a total score of up to 12. The disease is classified as remission (0–2), mild (3–5), moderate (6–10), or severe (11–12). **PGA**: physician global assessment, it is based on a 4-grade by an experienced gastroenterologist (SA) at baseline and follow-up 10–12 weeks after treatment escalation. The grades are 0 = clinical remission, 1 = mild disease activity, 2 = moderate disease activity, and 3 = severe disease activity. **UCEIS**: Ulcerative Colitis Endoscopic Index of Severity, an endoscopic index used to assess the severity of ulcerative colitis. It considers three variables: mucosal vascularization, bleeding, and ulceration/erosion, each scored from 0 to 3, resulting in a total score of 0 to 8. Inflammation is classified as minimal (0–1), moderate (2–4), or severe (5–8). **SES-CD**: Simple Endoscopic Score for Crohn’s Disease, an index used to assess the severity of Crohn’s disease based on endoscopic findings. It evaluates four parameters in each intestinal segment: extent of ulcerated lesions, extent of non-ulcerated lesions, ulcer size, and presence of strictures, with scores ranging from 0 to 3 for each criterion. The total score classifies the disease as mild (0–6), moderate (7–15), or severe (≥16). **CDEIS**: Segmental ileal and total Crohn’s disease endoscopic index of severity, similar to the SES-CD, it is an endoscopic index used to assess the severity of Crohn’s disease. It evaluates the extent of ulcers, deep inflammation, and the presence of strictures in different intestinal segments. The total score classifies the disease as remission (<3), mild (3–6), moderate (7–15), or severe (>15). **HBI**: Harvey-Bradshaw index, A simplified clinical index for assessing Crohn’s disease activity. It considers five criteria: general well-being, abdominal pain, number of liquid bowel movements per day, presence of complications, and abdominal mass. The total score classifies the disease as remission (<5), mild (5–7), moderate (8–16), or severe (>16). **SCCAI**: Simple Clinical Colitis Activity Index, a clinical index used to assess the activity of ulcerative colitis. It considers six parameters: daytime and nighttime bowel movement frequency, fecal urgency, rectal bleeding, general well-being, and extraintestinal manifestations. The total score classifies the disease as remission (≤2), mild (3–5), moderate (6–11), or severe (≥12).

Author	Objective	Participants	Diagnostic Method Activity	Sample
Rezaie et al. [10]	Determine whether salivary concentration of TGF-1 and NO might be helpful to evaluate or anticipate UC severity	UC group (n = 37) Mild activity (n = 21) Moderate activity (n = 8) Severe activity (n = 8) Control group (n = 15)	MTWSI	Unstimulated saliva
Szczeklik et al. [11]	Examine the prevalence of oral lesions in adult patients with CD and to investigate whether the salivary levels of interleukin 1β (IL-1β), IL-6, and TNF-α are associated with the activity and oral manifestations of CD	CD group (n = 95) Activity (n = 52) Mild activity (n = 14) Moderate activity (n = 38) Remission (n = 43) Control group (n = 45)	CDAI	Unstimulated saliva
Said et al. [7]	Compare the salivary microbiota of patients with IBD and healthy controls	CD group (n = 21) Remission (n = 13) Activity (n = 8) UC group (n = 14) Mild activity (n = 11) Moderate activity (n = 3) Control group (n = 24)	IOIBD and UC-DAI	Unstimulated saliva
Schaefer et al. [14]	Identify differentially expressed miRNAs that could selectively discriminate CD from UC and healthy controls using colon, blood, and saliva specimens	CD group (n = 42) UC group (n = 41) Control group (n = 35) * Only 5 saliva samples per group	Does not report	Unstimulated saliva
Zheng et al. [15]	Explore differences in the salivary protein contents of exosomes between patients with IBD and healthy subjects	CD group (n = 11) UC group (n = 37) Control group (n = 10)	Does not report	Unstimulated saliva
Szczeklik et al. [17]	Investigate the diagnostic usefulness of selected markers of oxidative stress in the serum and saliva of patients with active and inactive CD compared with healthy controls	CD group (n = 58) Activity (n = 32) Remission (n = 26) Control group (n = 26)	CDAI	Unstimulated saliva
Majster et al. [16]	Validate the analysis of calprotectin in saliva under several conditions, and to assess the levels in a small group of IBD patients with active disease, before and after treatment, compared to controls without bowel inflammation	CD group (n = 12) UC group (n = 11) Control group (n = 15)	PGA, UCEIS and SES-CD	Unstimulated and stimulated saliva
Buisson et al. [18]	Identify faster and less invasive tools to detect ileal colonization by adherent and invasive *E. coli* (AIEC) in patients with CD	CD group (n = 102)	CDEIS and CDAI	Does not report
Nijakowski et al. [12]	Determine how biologic drugs used in induction therapy would affect the salivary biochemical parameters and how these would be related to the clinical status in IBD patients	CD group (n = 27) UC group (n = 24)	CDAI and the modified Mayo scale	Unstimulated saliva
Nijakowski et al. [13]	Compare salivary concentrations of selected biomarkers in patients with Crohn’s disease and ulcerative colitis to determine whether they could be of predictive value for the differential diagnosis	CD group (n = 27) UC group (n = 24) Control group (n = 51)	CDAI and the modified Mayo	Unstimulated saliva
Bos et al. [19]	Explore if salivary calprotectin could be used as a reliable non-invasive biomarker in IBD	CD group (n = 42) Remission (n = 34) Activity (n = 5) Missing (n = 3) UC group (n = 21) Remission (n = 15) Activity (n = 2) Missing (n = 4) Control group (n = 11)	HBI score or SCCAI	Stimulated saliva
Elzayat et al. [20]	Characterize the compositional changes in the salivary microbiota of patients with CD compared to healthy controls	CD group (n = 40) Activity (n = 10) Remission (n = 30) Control group (n = 40)	CDAI	Unstimulated saliva

The “*” indicates the number of saliva samples used, as it differs from the number of study participants.

As shown in Table 1, seven studies analyzed individuals with CD and UC [7,12,13,14,15,16,19], while four focused only on CD [11,17,18,20] and one on UC [10]. Furthermore, nine studies used unstimulated saliva [7,10,11,12,13,14,15,17,20], while one used stimulated saliva [19] and another used both [16]. However, one study did not mention the type of saliva used [18]. Most studies used the CDAI index to classify disease activity in individuals with CD [11,12,13,17,18,20]. However, there was no prevalent index for UC; instead, various methods were used, such as the modified Mayo scale [12,13], UC-DAI [7], Truelove-Witts’s severity index, modified Truelove-Witts’s severity index (MTWSI) [10], and Simple Clinical Colitis Activity Index (SCCAI) [19]. In some cases, the index was either not reported or not relevant to the study [14,15].

### 3.4. Methodology Employed in Each Study

Table 2 summarizes the key findings from the 12 selected studies, focusing on the identification of molecules and/or proteins present in saliva. It also summarizes the methodological design of each study, including classification by research approach (e.g., case–control, prospective study).

Seven studies were classified as case–control [7,10,14,15,16,17,20]. Two studies prospective cohort [12,13] and three studies were classified as prospective study, multicenter prospective, and exploratory cross-sectional cohort [11,18,19], respectively.

Eight studies conducted immunoassays and enzymatic assays for molecules analysis, such as ELISA kits, to identify TGF-β1, NO [10], IL-1β, IL-6, TNF-α [11], LL-37 [7], calprotectin [12,13,16,20], CEACAM6 [18], TNF-R1, Serpin E1/PAI-1, myeloperoxidase, IgA, Catalase [12,13] and C-reactive protein [20]. Two studies used turbidimetric immunoassay [7,19], while one study conducted MicroRNA analysis [14]. Three studies performed total protein quantification using the Bradford method [7,12,13], and one used the bicinchoninic acid (BCA) method [17]. One study conducted proteomic analysis [15], one analyzed the microbiome [20], one conducted bacterial 16S rRNA gene-based analysis [7], another used Luminex Fluorescence Technique, IgA testing [7], while other studies applied Western blotting [15] and spectrophotometry [17]. Unlike ELISA, spectrophotometry quantifies molecules by measuring light absorbance based on their optical properties, without requiring antibodies [21].

Szczeklik and colleagues [11] considered the prevalence of oral lesions in individuals with CD, such as polyploid tag lesions, cobble stoning, buccal swelling, gingivitis, deep ulcers, aphthous ulcers, angular cheilitis, atrophic glossitis, coated tongue, median lip fissure, lymphadenopathy, with respect to the presence of some molecules in saliva. The study by Buisson and colleagues [18] investigated a direct relationship between ileal colonization by adherent-invasive *E. coli* (AIEC) and CD in patients.

Six studies used saliva samples only [7,10,11,12,13,20]. The study by Schaefer and colleagues [14] used colonic mucosa biopsies, blood and saliva, with only 5 saliva samples per group (5 CD, 5 UC, 5 Control). Three studies used saliva and blood [16,17,19], with the study by Bos and colleagues [19] also using stool. The study by Zheng and colleagues [15] primarily used saliva for most analyses, but briefly mentioned that an animal study was also conducted. In contrast, the study by Buisson and colleagues [18] used ileal biopsy, stool, saliva and blood.

## 4. Discussion

In this scoping review, various techniques were employed for the identification and analysis of molecules and/or microorganisms, including assays such as ELISA, as well as high-throughput approaches such as genomics, proteomics, and microbiome analysis (or metagenomics). Genomics involves the study of complete genomes [1]. Proteomics seeks to determine the set of proteins (the proteome) present in a sample [1,5]. This analysis is frequently performed in conjunction with separation and detection techniques, such as liquid chromatography, gel electrophoresis, and mass spectrometry [1,5]. Microbiome analysis seeks to identify all microorganisms present in the sample, which generally involves DNA/RNA extraction, followed by analyses based on the bacterial 16S rRNA gene [1,5]. Furthermore, it is important to classify these methodologies based on their analytical strengths, with omics techniques offering high discovery potential for identification [1,5,22]. In contrast, ELISA offers high quantitative power, for example, the validation and measurement of specific molecules [1,5,22]. Thus, mapping (Figure 2) these methodologies is crucial for the correct interpretation and presentation of results found in the literature.

### 4.1. Geographical Visualization and Spatial Analysis of the Included Studies Based on the Evidence Map

At the beginning of the 21st century, the epidemiological pattern of IBD changed significantly [23,24]. While the incidence rate stabilized or declined in regions such as North America, Europe and Australia, newly industrialized countries in Africa, Asia and South America experienced a notable increase [23,24]. The prevalence of the disease continues to grow in regions where it has historically been prevalent, with rates exceeding 0.3% of the population in countries such as Canada, Denmark, Germany, Hungary, Australia, New Zealand, Sweden, the United Kingdom and the United States [23].

Notably, a considerable number of the studies included in this review were conducted in Poland [11,12,13,17]. This finding can be attributed to the increased prevalence of the disease, with 253 cases per 100,000 inhabitants in 2020, stimulating research into less invasive diagnostic methods [25]. Interest in the oral-systemic connection (‘gum-gut axis’) and the existence of national databases for large-scale population studies also contribute to Poland’s leadership in this area [24,25,26,27,28].

This geographic disparity is critical when interpreting biomarker results, as the profile of salivary molecules, notably the microbiome, can be population-dependent and influenced by local factors such as genetics, diet, and regional environmental exposures [12,23,24]. Therefore, the lack of studies from newly industrialized regions represents a significant gap, limiting the generalizability of current findings.

The processes of industrialization and urbanization in certain regions have led to a rising incidence and prevalence of IBD [23,24], highlighting the need for further research aimed at supporting public health strategies and addressing the growing burden of disease in each country.

### 4.2. Schematic of the Relationship Between Oral and Intestinal Health

**Figure 4:**
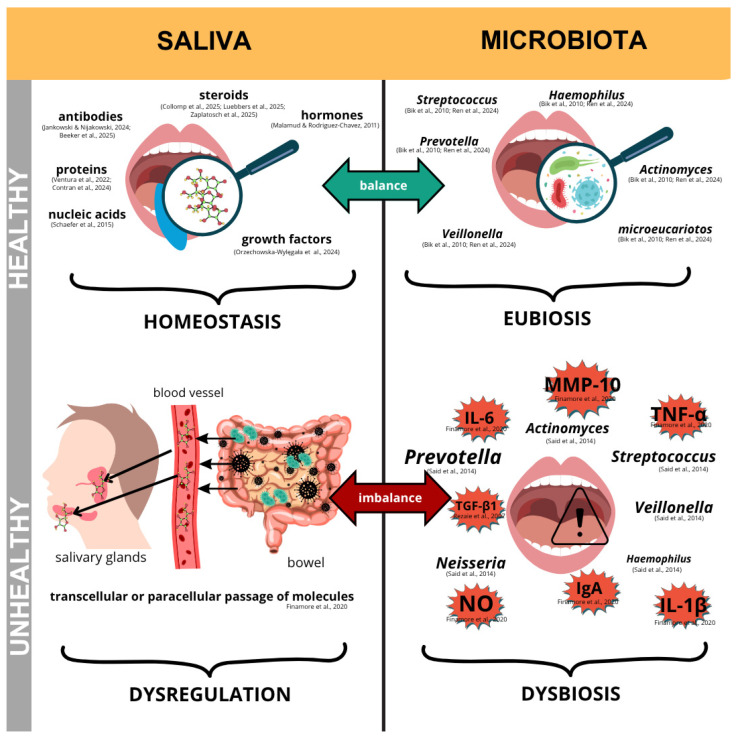
Scheme of the representation of changes in the oral region due to the influence of IBD [4,6,7,10,30,31,32,33,34,35,36,37,38,39].

Saliva is mainly composed of water, but also contains electrolytes, proteins, hormones, metabolites, and other molecules, as well as mucosal cells and microorganisms [29]. Saliva can be classified as stimulated, produced in response to mechanical or gustatory stimulation, or unstimulated, typically collected from individuals at rest [29]. Due to its complex composition, saliva can reflect both the state of the oral cavity and systemic conditions, as illustrated in Figure 4.

Figure 4 shows the salivary profile and oral microbiota in healthy individuals. Changes in saliva can lead to dysbiosis in the presence of IBD, thereby increasing susceptibility to periodontal disease or oral lesions, with disease-associated molecular changes (cytokines, hormones, proteins, etc.) contributing to this imbalance.

Under conditions of microbial balance, referred to as eubiosis, the healthy oral cavity presents a complex composition of metabolites in oral fluids, including immunoglobulins, antibodies, hormones, and growth factors (Figure 4) [4,7,14,30,31,32,33,34,35]. In this environment, the oral microbiota is predominantly composed of bacteria from the *Streptococcus* genus, although proteobacteria can also be identified [36,37]. However, inflammatory and/or chronic systemic conditions may promote dysbiosis, altering the microbial community and salivary components [36,37]. During saliva formation, transcellular and paracellular transport of blood-derived metabolites occurs, allowing the detection of circulating disease biomarkers [6].

In IBD, bacterial metabolites produced by the gut microbiota stimulate systemic immune responses, resulting in the release of anti-inflammatory mediators that circulate between the intestinal epithelium and blood vessels (as illustrated in Figure 4). This systemic interaction can alter the salivary composition and modulate the expression of specific proteins, such as MMP-10, IL-6, IL-1β, and TGF-β1; and IgA, a key mucosal immunoglobulin with anti-inflammatory functions [6,7]. Simultaneously, the increase prevalence of genera such as *Prevotella*, *Veillonella*, and *Neisseria*, and the reduction in *Haemophilus* and *Streptococcus* species establish dysbiosis, an imbalance in the different microorganisms living together in a microbiome [6,7].

Despite ongoing efforts, whether these salivary changes are a consequence of IBD or precede and contribute to the disease and its development remains unclear [7]. In other words, the causal relationship between salivary profile alterations and the onset of IBD has yet to be clearly established [7].

### 4.3. Oral Health

The present scoping review demonstrates new technologies for analyzing metabolic or microbiota saliva in patients with CD and UC, synthesized through the evidence mapping in Figure 2.

Oral health is a crucial factor, as the presence of dental caries, gingivitis or periodontal disease (PD) can alter microbiota and metabolite levels, thereby influencing outcomes associated with IBD [8,20]. Unlike reversible gingivitis [40], PD destroys the tissues that support the teeth and can lead to tooth loss [8,40,41,42]. The progression of PD involves an interaction between pathogens and the host’s immune response, which is similar to what occurs in IBD [8,42]. In both diseases, dysbiosis produces bacterial components that induce an inflammatory response with increased cytokines such as IL-1β, IL-6, TIMP-3, and MMPs [8,43,44]. Considering these characteristics, some studies have shown an increased prevalence of PD in patients with IBD [45,46].

Regarding the measurement of PD, the study conducted by Habashneh and colleagues [45] included individuals diagnosed with IBD for at least one year. A comprehensive periodontal examination was performed to assess the presence of PD, including measurements of probing depth, as well as the assessment of plaque index, gingival index, and dental calculus. These parameters were then compared between the IBD group and the control group. Similarly, the study by Zilberstein and colleagues [46] applied the same periodontal evaluation, documenting bone probing depth, attachment loss, plaque index, gingival index, and bleeding index, while also comparing these findings to a control group.

These observations corroborated other works [7,20], which identified altered oral microbiome in patients with IBD, such as a lower Firmicutes/Bacteroidetes (F/B) ratio, an indicator of homeostasis [20,47], as well as an increase in opportunistic bacterial species [20], such as *Dolosigranulum pigrum* and *Prevotella jejuni* [48,49,50] in CD patients (Table 2).

Furthermore, the study by Elzayat and colleagues [20] detected significant species-level characteristics in patients with CD with different oral health status. Namely, patients with poor oral health presented high levels of *Porphyromonas gingivalis*, *Fusobacterium periodonticum*, *Lactobacillus fermentum*, *Lactobacillus acidophilus*, and *Streptococcus mutans*, whereas those with good oral health and CD also showed high levels of *P. gingivalis*, *Prevotella jejuni*, *Prevotella dentalis*, *Tannerella forsythia*, and *Bacteroides fragilis*. Many of these are pathogenic or opportunistic pathogens, indicating that oral dysbiosis may occur regardless of the oral condition in patients with CD.

The study by Said and colleagues [7] also showed an increase in the genus *Prevotella* in the salivary microbiota of patients with IBD, similarly to what occurs in conditions such as esophagitis [51] and active dental caries [52], alteration in some salivary proteins are observed, including an increase in IgA and a decrease in lysozyme levels. However, a limitation of this study was the lack of access to oral health clinical records, as elevated IgA levels may reflect both IBD and inflammatory oral lesions [7,8,53], and reduced salivary lysozyme level can also be presented in cases of gingivitis and periodontitis [54].

Another study conducted oral examination, but it had excluded patients with PD due to the similarity in the increase in certain cytokines (IL-6, y IL-1β, TNF-α) in patients with IBD and those with PD [11], hindering the distinction between both conditions. Conducting oral examinations is crucial, since lesions other than periodontitis, such as indurated polypoid tag lesions, diffuse swellings, focal areas of mucosal inflammatory hyperplasia, fissuring and Granulomatous cheilitis, classified according to Scheper and Brand and colleagues [55], appear in patients with both active and inactive CD. Additionally, salivary IgA levels may be elevated in patients with such oral mucosal lesions, regardless of the presence and activity of CD [56,57].

Therefore, oral parameters are essential for distinguishing biochemical changes related to oral conditions from those associated with systemic diseases like CD and UC [7,11,12,20]. Based on this, determining whether the molecular findings of the studies selected in this review are consistent with a systemic disease, oral disease, or both.

### 4.4. Intestinal Barrier Integrity and Microbiota

Once the influence of oral lesions has been considered, understanding how metabolites and microorganisms manifest themselves in CD and UC becomes crucial. Intestinal dysbiosis, which is an alteration in the microbiota/host relationship, stimulates intestinal inflammation in both conditions [58]. In CD, for example, this includes the presence of adherent and invasive *E. coli* (AIEC), whereas in UC, there is an increase in *Oscillibacter* and *Ruminiclostridium 6* [18,59]. Intestinal barrier dysfunction, induced by inflammatory cytokines such as IFNγ and TNFα, contributes to increased permeability [59,60], as illustrated in Figure 4. Consequently, the intestinal microbiome may serve as a potential biomarker [59].

**Figure 5:**
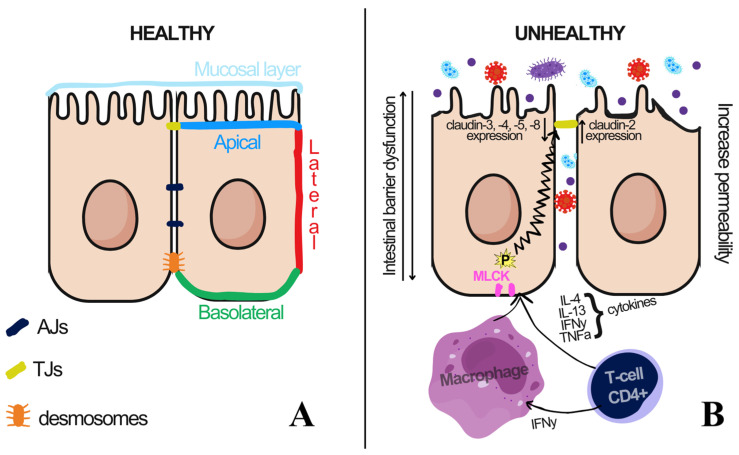
The intestinal barrier. (**A**) simplified anatomy of intestinal epithelial cells, which consists of three structures: adherent’s junctions (AJs), tight junctions (TJs), and desmosomes. (**B**) simplified diagram of intestinal barrier dysfunction induced by cytokines such as IFNγ, IL-10, MLCK, and TNFα (derived from CD4+ T cells and macrophages), as well as IL-4 and IL-13. This increase in cytokines can lead to the disruption of TJs, specifically affecting claudins 3, 4, 5, and 8 while increasing claudin-2 expression, resulting in enhanced permeability. Adapted from [60].

The intestinal epithelium acts as a barrier and selective filter (Figure 5), regulating permeability through two pathways: transepithelial (solute transport) and paracellular (transport between cells) [61]. The interaction between epithelial cells occurs through adherent’s junctions (AJs), tight junctions (TJs), and desmosomes [60,62] (Figure 5). An imbalance in the expression of claudins and occludins is shown in IBD [60,63].

Intestinal inflammation observed in patients with IBD is largely driven by mucosal colonization by AIEC [59,64]. More specifically in CD, this colonization induces the activation of intestinal T cells, leading to the release of pro-inflammatory cytokines, such as IFNγ and TNFα [60]. However, identifying patients with CD colonized by AIEC represents a clinical challenge, as characterizing bacterial strains with adherent and invasive properties requires specific methods [18]. In this context, the study by Buisson and colleagues [18] proposed alternative approaches for this identification, such as detection of anti-E. coli antibodies (AEcAb), as shown in Table 2. Additionally, the same study revealed that the adhesion molecule CEACAM6, which acts as a receptor for AIEC in intestinal epithelial cells [65], showed a significant association with the total *E. coli* load (*p* = 0.028). Moreover, CEACAM6 levels exhibited a positive correlation with CEACAM6 values in both saliva and the ileum, reinforcing its potential role in the pathophysiology of CD associated with AIEC colonization.

One indicator of intestinal inflammation is an increase in fecal calprotectin [16]. During an active disease, calprotectin levels could indicate neutrophil migration to the intestinal mucosa [16], highlighting fecal calprotectin as an important non-invasive biomarker for monitoring intestinal inflammation and disease activity in patients with IBD. However, its presence in saliva remains debatable [16,19,20].

### 4.5. Salivary Parameters—Oxidative Stress

Oxidative stress has been extensively studied in IBD, as the excessive production of reactive oxygen species and the reduction in antioxidant mechanisms may contribute to the pathogenesis of these conditions [16,66]. The study by Szczeklik and colleagues [17] evaluated lipid peroxidation indicators and antioxidant status in the serum and saliva of patients with active CD, observing a significant increase in oxidative stress markers such as malondialdehyde (MDA) and a reduction in antioxidant levels, including superoxide dismutase (SOD), and glutathione peroxidase (GSH) (Table 2). These findings suggest an imbalance between free radical production and antioxidant defense in patients with active CD.

Another study identified potential salivary biomarkers in UC, including nitric oxide (NO), which was found at levels four times higher than normal values, suggesting systemic dysregulation of NO production [10]. NO plays a crucial role in maintaining a balance between physiological, pro-inflammatory, and cytotoxic levels [10,67]. Notably, Rezaie and colleagues [10] evaluated the periodontal inflammation status of participants; however, the methodology employed to ascertain this information remains unclear.

These findings underscore the importance of saliva as an accessible and promising sample for assessing oxidative stress in IBD and its potential application in monitoring disease activity.

### 4.6. Salivary microRNA

A new class of RNAs, miRNAs (a single-stranded RNA molecule of 19–25 nucleotides), are small non-coding RNA molecules with a crucial role in the regulation of gene expression, acting at a stage before protein synthesis [68]. They may contribute to defining the etiology and pathology of IBD, as they are involved in various functions such as differentiation, organogenesis, and metabolism [69].

The study by Schaefer and colleagues [14] identified a specific set of differentially expressed miRNAs between these diseases in colonic mucosa biopsies, blood, and saliva, suggesting potential diagnostic biomarkers. Among the saliva findings, an increase in miR-101 in the CD group compared to the control was observed, as well as an increase in miR-21, miR-31, and miR-142-3p in the UC group, along with a reduction in miR-142-5p in the UC group compared to the control (Table 2). MiR-21 is known to destroy tight junctions [70], miR-31 is present in inflammation and cancer [71], miR-101 is anti-proliferative (tumor suppressor via gene regulation) [12,72], whereas miR-142-3p regulates the formation and differentiation of hematopoietic stem cells [73] and miR-142-5p is a negative regulator of TGF-β [74]. It is essential to highlight that the microRNA analysis was performed on five individuals from each group. These results reinforce the importance of miRNA analysis as a promising tool for differentiating IBD subtypes and monitoring disease progression.

MicroRNA analysis is a relatively recent and innovative approach in molecular biology. By influencing mRNA stability and translation, their evaluation enables the identification of early molecular alterations, potentially preceding changes observed at the proteomic level [69,70], contributing to a deeper understanding of the biological pathways involved, and supporting the identification of novel diagnostic or prognostic biomarkers [69,75].

### 4.7. Other Promising Inflammatory Molecules in IBD

The current scientific community seeks to understand the oral-systemic axes to improve oral and gastrointestinal health [76]. Saliva is the gateway to the gastrointestinal tract and transports enzymes, cytokines and inflammatory cells on a daily basis [3]. These substances travel through the GI tract, as the mucus produced in the mouth provides protection against gastric acid [3,76]. Studies aimed at understanding these pathways are of fundamental importance.

For example, the study by Rezaie and colleagues [10] reported an increase in salivary TGF-β1 levels in patients with UC (Table 2). TGF-β1 is typically present near the secreting cell or adjacent cells [77] and one of its functions is to stimulate the cellular differentiation of epithelial cells during the repair processes in damaged mucosa [10,77]. However, no difference in TGF-β1 levels has been found among patients with UC at different disease activity stages [78].

The study by Said and colleagues [7] demonstrated an increase in salivary cytokines in individuals with UC. Furthermore, lysozyme levels were found to be reduced when comparing the IBD and control groups, while IgA and LL-37 levels were increased in the IBD group (Table 2). The higher levels of IgA suggested that patients with IBD may have oral manifestations, but this study did not have access to the patients’ oral cavity. On the other hand, the studies by Nijakowski and colleagues [12,13] showed that the levels of myeloperoxidase and IgA were reduced in patients with UC and CD compared to healthy controls. However, it is important to note that this study also considered the type of treatment and its timing. During the induction phase of biological therapy, a significant increase in these markers was shown in patients with UC who responded to treatment (Table 2), suggesting that biological therapy may restore the host’s oral defense, which was previously compromised by the disease’s active state and the immunosuppression often associated with conventional treatment, such as corticosteroids [11,17].

Another study, using exosomal proteins, found that PSMA7 (proteasome subunit alpha type 7) was elevated in individuals with IBD compared to the control group [15] (Table 2). This protein is involved in inflammatory responses, such as the regulation of protein degradation and various cellular pathways (Uniprot). Whereas lysozyme, an antimicrobial protein able to catalyze the hydrolysis of Gram-positive bacterial cell wall [54] (Uniprot) and exhibit bactericidal activity against Gram-negative bacteria, is reduced in patients with IBD [7]. This finding suggests that lysozyme reduction may be associated with the change in oral microbiota, since high prevalence of *Streptococcus*, *Prevotella*, *Veillonella*, and *Haemophilus* was also found in patients with IBD [7]. However, it is important to note that the cited study [7] did not include a clinical oral examination to further investigate this relation.

Despite being used in routine clinical practice, fecal calprotectin still shows contrasting results in saliva, and further studies are needed [13,16,19].

### 4.8. Summary of Findings

**Figure 6:**
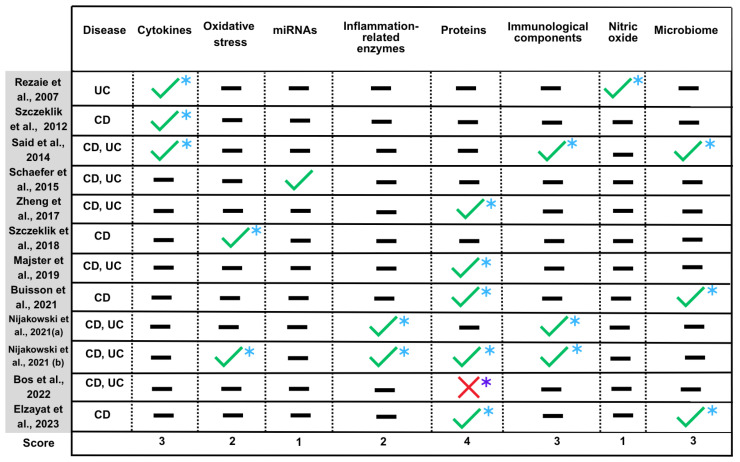
Synthesis of the findings about salivary biomarkers related to IBD. The definition of all symbols (✓, X, —, *) and the methodology used for calculating the final Score are detailed in the Materials and Methods Section (Section 2.5. Quantified synthesis and categorization of biomarkers) [7,10,11,12,13,14,15,16,17,18,19,20].

Figure 6 provides a simplified summary of the molecules with potential—or lack thereof—to act as biomarkers in the diagnosis, monitoring or therapeutic selection in Crohn’s disease and ulcerative colitis.

A body of research has already been conducted on the potential of biomarkers such as IL-1β, IL-6, TNF-α, and calprotectin to inform clinical decisions [6]. It should be noted, however, that the study by Bos and colleagues [19] showed no promising results regarding the calprotectin protein, which is why it was marked with “X” and “*” in Figure 6. These biomarkers have demonstrated strong diagnostic and prognostic value, suggesting their potential for use for disease monitoring and management [6,12]. Evidently, other markers, including certain microRNAs, immunological components, and oxidative stress-related metabolites, demonstrate significant potential [6,14,46,67]. However, further studies are required to provide definitive validation. On the other hand, conditions such as PD and dental caries, although prevalent in individuals with IBD, are considered confounding factors and, therefore, data interpretation should be cautious [8,36,46].

This scoping review identified important findings regarding different biomarkers. However, some limitations need to be mentioned, such as the small number of studies, as well as their quality (case–control, prospective studies, etc.), and new techniques being improved, such as proteomics. Therefore, future research should focus on implementing standardized protocols, performing large-scale validation studies, and increasing the methodological robustness of comparative assays to allow for clinical translation of these preliminary findings.

## 5. Conclusions

This scoping review demonstrates the promising potential of salivary biomarkers for diagnosing and monitoring both Crohn’s disease (CD) and Ulcerative Colitis (UC), despite current methodological heterogeneity. The identification of sensitive and specific biomarkers in this cost-effective, non-invasive matrix is essential for developing a practical, saliva-based ‘IBD panel.’ To achieve this potential, future research must focus on standardizing study protocols, particularly regarding oral cavity pre-assessment and saliva collection techniques. Crucially, prospective cohort studies with larger sample sizes are necessary for the validation of the most promising molecules, ultimately enabling the clinical integration of salivary data for enhanced IBD management.

## Figures and Tables

**Table 2:** Methodology and main results of the 12 selected studies. This table presents the key outcomes of the studies included in the scoping review, detailing the study design, analytical methods used, specific molecules and/or microorganisms investigated in saliva, and mean results reported, including observed alterations and correlations. Abbreviations and notations: ↔: without alteration or correlation. ↑: increase. ↓: decrease. CC: case–control. PS: prospective study. PC: prospective cohort. ECSC: exploratory cross-sectional cohort. MP: Multicenter prospective. TGF-β1: Transforming growth factor beta 1. NO: nitric oxide. IL-1β: Interleukin 1 beta. IL-6: interleukin 6. TNF-α: Tumor necrosis factor alpha. IBD: inflammatory bowel disease. LL-37: cathelicidin-derived antimicrobial peptide. IgA: immunoglobulin A. PSMA7: Proteasome subunit alpha type-7. MDA: malondialdehyde. FRAP: ferric reducing ability of plasma. GSH: reduced glutathione. CAL: calprotectin. AIEC: adherent-invasive Escherichia coli. CEACAM6: cell adhesion molecule 6. MPO: Myeloperoxidase. TNF-R1: Tumor necrosis factor receptor-1. PAI-1: Plasminogen activator inhibitor-1. CRP: C-reactive protein.

Authors	Design	Analysis	Molecules/Microorganisms in Saliva	Mean Results
Rezaie et al. [10]	CC	ELISA	TGF-β_1_, NO	- ↑ TGF-β1 and NO (*p* > 0·05) - NO ↔ TGF-β1 (no corr., *p* = 0.74)
Szczeklik et al. [11]	PS	ELISA Oral examinations	IL-1β, IL-6, TNF-α	- ↔ salivary flow - ↑ IL-1β, IL-6, TNF-α (active > inactive & ctrl; *p* < 0.05) - IL-1β, IL-6, TNF-α ↔ (inactive CD vs. ctrl) - ↑ IL-6, TNF-α (saliva + lesions, active CD; *p* < 0.05); IL-1β ↔ (*p* = 0.282)
Said et al. [7]	CC	Barcoded 16S rRNA pyrosequencing Immunoassays	Microbiota, cytokines, LL-37, IgA, Salivary lysozyme, total protein concentration	- ↑ *Bacteroidetes* and ↓ *Proteobacteria* in CD and UC vs. control (*p* < 0.01; *p* < 0.05) - ↔ Phylum level: UC vs. CD - ↑ *Prevotella* (phy. Bacteroidetes) and *Veillonella* (phy. Firmicutes): IBD vs. control (*p* < 0.01) - ↓ *Streptococcus* and *Haemophilus*: IBD vs. control (*p* < 0.05) - Gram-positive vs. Gram-negative ↔ (among all groups) - Lysozyme ↓ (IBD vs. ctrl; *p* < 0.01); IgA, LL37 ↑ (IBD vs. ctrl; *p* < 0.05)
Schaefer et al. [14]	CC	Microarrays Isolation of RNA and real-time quantitative PCR (qRT-PCR)	miRNA and potential miRNA target genes	- miR-101, miR-21, miR-31, miR-142-3p ↑ (IBD vs. ctrl; *p* < 0.05) - miR-142-5p ↓ (UC vs. ctrl; *p* < 0.05) - miR-101 → potential key regulator in IBD
Zheng et al. [15]	CC	Extraction of exosomes from saliva Western blotting Shotgun mass spectroscopy analysis	Exosomal proteins and PSMA7 protein	- 8 proteins present only in CD and UC - PSMA7 ↑ (CD and UC vs. ctrl) - PSMA7 ↓ (remission vs. active disease)
Szczeklik et al. [17]	CC	Colorimetric method based on thiobarbituric acid (TBA) reactivity FRAP method Ellman method	MDA, GSH, FRAP levels	- MDA ↑ (active CD vs. inactive CD and ctrl; *p* < 0.01) - FRAP ↓ (CD vs. ctrl) - GSH ↓ (active CD vs. inactive CD and ctrl; *p* < 0.01)
Majster et al. [16]	CC	Immunoassays	Calprotectin levels (CAL)	- CAL higher in stimulated vs. unstimulated saliva (fasting and non-fasting; *p* < 0.001) - CAL 4.0-fold ↑ in stimulated saliva of IBD patients (*p* = 0.001) - CAL ↑ in CD vs. ctrl (unstimulated *p* = 0.011; stimulated *p* = 0.002) - CAL ↑ in UC vs. ctrl (stimulated saliva; *p* = 0.021) - Salivary CAL higher in ileal CD and treatment-naïve patients; no correlation with disease extension
Buisson et al. [18]	MP	*E. coli* counting and identification Invasion assay ERIC-PCR Anti-E. coli antibody measurement and quantification CEACAM6 by ELISA	CEACAM6 levels	- AIEC colonized ileum in 24.5% of CD patients (25/102) - Global invasive ability of ileal total *E. coli* ↑ in AIEC-positive vs. AIEC-negative patients (*p* = 0.0007) - Salivary CEACAM6 positively correlated with ileal CEACAM6 (healthy areas *p* < 0.0001; ulcerated zones *p* = 0.0082; overall *p* < 0.0001) - Salivary CEACAM6 levels not different in AIEC-positive vs. AIEC-negative patients (*p* = 0.45)
Nijakowski et al. [12]	PC	Enzyme-linked immunosorbent assays Bradford method	Myeloperoxidase (MPO), immunoglobulin A (IgA) and total protein levels	- pH and stimulated flow ↑ in CD and UC - No difference in salivary flow rate, IgA, or MPO between CD patients with successful vs. unsuccessful therapy - IgA and MPO ↑ in UC responders to biological therapy (*p* = 0.009 and *p* = 0.004, respectively)
Nijakowski et al. [13]	PC	Immunoassays and enzymatic colorimetric assays Bradford method	IgA, S100A8/calprotectin, TNF-R1, PAI-1, MPO, catalase, total protein levels	- IgA, CAL, MPO ↓ in CD and UC (*p* < 0.05) - TNF-R1, catalase ↓ in UC (*p* < 0.05) - Salivary protein concentration ↓ in IBD vs. ctrl (*p* < 0.001) - PAI-1 similar across all groups
Bos et al. [19]	ECSC	Particle-enhanced turbidimetric immunoassay	Calprotectin (CAL)	- No significant correlation: salivary CAL vs. fecal CAL (*p* = 0.495) and salivary CAL vs. plasma CP (*p* = 0.223) - No significant difference in salivary CAL between active disease and remission
Elzayat et al. [20]	CC	CRP and CAL concentrations were determined by ELISA kits Microbiome	Microbiota, CAL, C-reactive protein (CRP)	- CD: salivary CRP, CAL ↑ vs. ctrl, non-significant - Salivary CAL ↑ in caries vs. periodontal disease (*p* = 0.009); CRP no significant difference (*p* > 0.05) - Five species ↑ in CD vs. ctrl: *Veillonella dispar*, *Prevotella jejuni*, *Dolosigranulum pigrum*, *Lactobacillus backii*, *Megasphaera stantonii* - Phyla level: *Fusobacteria* in CD with good oral health (H); *Actinobacteria* in periodontal disease (P) - Genus level: *Bacteroides* (H), *Streptococcus* (P), *Fusobacteria* (CD + caries, C), *Lactobacillus* (P + C) - Species level: H: *Neisseria subflava*, *Tannerella forsythia*, *Porphyromonas gingivalis*, *Prevotella jejuni*, *P. dentalis*, *P. enoeca*, *Bacteroides fragilis*, *B. intestinalis*; P:*. mutans*, *S. pyogenes*, *S. oralis*, *S. viridans*; P + C: *S. mutans*, *L. fermentum*, *L. acidophilus* *- Simonsiella dominant* in CD patients treated only with monoclonal antibodies *- Simonsiella muelleri* exclusive to monoclonal antibody therapy; *E. coli*, *S. enterica* ↑ in triple therapy - Genus level: *Porphyromonas* ↑ in newly diagnosed; *Pasteurella* ↑ in long-term CD - *Klebsiella pneumoniae* detected in CD > 10 years - Genus level: *Acetoanaerobium*, *Mycoplasma* ↑ in active CD; *Schaalia*, *Cardiobacterium*, *Leptotrichia*, *Capnocytophaga* ↑ in inactive CD - Frequent relapsers: *Prevotella* spp., *Simonsiella muelleri*; infrequent: *Clostridium*, *Lactobacillus*, *Ruminococcus* - Seven oral species overlapped with IBD medications and oral health, including *L. jensenii*, *E. durans*, and *A. pittii*.

## Data Availability

No new data were created or analyzed in this study. Data sharing does not apply to this article.

## Abbreviations

The following abbreviations are used in this manuscript:

IBD	Inflammatory bowel disease
CD	Crohn’s disease
UC	Ulcerative colitis
MPO	Myeloperoxidase
NO	Nitric oxide
ELISA	Enzyme-linked immunosorbent assay
IgA	Immunoglobulin A
TNF-R1	Tumor Necrosis Factor Receptor Type 1
PAI-1	Plasminogen Activator Inhibitor-1
miRNA	microRNA
CEACAM6	Carcinoembryonic Antigen Related Cell Adhesion Molecule 6
IL-1β	Interleukin IL-1β
IL-6	Interleukin 6
TNF-α	Anti-tumor necrosis factor α
MDA	Malondialdehyde
GSH	Glutathione
FRAP	Ferric reducing ability of plasma
LL-37	Cathelicidin-derived antimicrobial peptide
TGF-β1	Transforming Growth Factor-β1
NO	Nitric Oxide
CRP	C-reactive protein
MTWSI	Modified Truelove-Witts severity index
CDAI	Crohn’s disease activity index
IOIBD	International Organization of Inflammatory Bowel Disease index
UC-DAI	Ulcerative Colitis Disease Activity Index
PGA	Physician global assessment
UCEIS	Ulcerative Colitis Endoscopic Index of Severity
SES-CD	Simple Endoscopic Score for Crohn’s Disease
CDEIS	Segmental ileal and total Crohn’s disease endoscopic index of severity
HBI	Harvey-Bradshaw index
SCCAI	Simple Clinical Colitis Activity Index
PSMA7	Proteasome subunit alpha type-7
CAL	Calprotectin
AIEC	Adherent-invasive *Escherichia coli*
SOD	Superoxide dismutase
PRISMA-ScR	Systematic reviews and Meta-Analyses extension for Scoping Reviews
OSF	Open Science Framework
